# Comparative evaluation of ^18^F-FLT and ^18^F-FDG for detecting cardiac and extra-cardiac thoracic involvement in patients with newly diagnosed sarcoidosis

**DOI:** 10.1186/s13550-017-0321-0

**Published:** 2017-08-29

**Authors:** Takashi Norikane, Yuka Yamamoto, Yukito Maeda, Takahisa Noma, Hiroaki Dobashi, Yoshihiro Nishiyama

**Affiliations:** 1 Department of Radiology, Faculty of Medicine, Kagawa University, 1750-1 Ikenobe, Miki-cho, Kita-gun, Kagawa 761-0793 Japan; 2 Division of Cardiorenal and Cerebrovascular Medicine, Department of Internal Medicine, Faculty of Medicine, Kagawa University, Kita-gun, Kagawa Japan; 3 Division of Hematology, Rheumatology and Respiratory Medicine, Department of Internal Medicine, Faculty of Medicine, Kagawa University, Kita-gun, Kagawa Japan

**Keywords:** ^18^F-FLT, ^18^F-FDG, PET, Sarcoidosis

## Abstract

**Background:**

^18^F-FDG PET has been used in sarcoidosis for diagnosis and determination of the extent of the disease. However, assessing inflammatory lesions in cardiac sarcoidosis using ^18^F-FDG can be challenging because it accumulates physiologically in normal myocardium. Another radiotracer, 3′-deoxy-3′-^18^F-fluorothymidine (^18^F-FLT), has been investigated as a promising PET tracer for evaluating tumor proliferative activity. In contrast to ^18^F-FDG, ^18^F-FLT uptake in the normal myocardium is low. The purpose of this retrospective study was to compare the uptake of ^18^F-FLT and ^18^F-FDG in the evaluation of cardiac and extra-cardiac thoracic involvement in patients with newly diagnosed sarcoidosis.

Data for 20 patients with newly diagnosed sarcoidosis were examined. ^18^F-FLT and ^18^F-FDG PET/CT studies had been performed at 1 h after each radiotracer injection. The patients had fasted for at least 18 h before ^18^F-FDG PET/CT but were given no special dietary instructions regarding the period before ^18^F-FLT PET/CT. Uptake of ^18^F-FLT and ^18^F-FDG was examined visually and semiquantitatively using maximal standardized uptake value (SUVmax).

**Results:**

Two patients had cardiac sarcoidosis, 7 had extra-cardiac thoracic sarcoidosis, and 11 had both cardiac and extra-cardiac thoracic sarcoidosis. On visual analysis for diagnosis of cardiac sarcoidosis, 4/20 ^18^F-FDG scans were rated as inconclusive because the ^18^F-FDG pattern was diffuse, whereas no FLT scans were rated as inconclusive. The sensitivity of ^18^F-FDG PET/CT for detection of cardiac sarcoidosis was 85%; specificity, 100%; and accuracy, 90%. The corresponding values for ^18^F-FLT PET/CT were 92, 100, and 95%, respectively. Using semiquantitative analysis of cardiac sarcoidosis, the mean ^18^F-FDG SUVmax was significantly higher than the mean ^18^F-FLT SUVmax (*P* < 0.005). Both ^18^F-FDG and ^18^F-FLT PET/CT studies detected all 24 extra-cardiac lesions. Using semiquantitative analysis of extra-cardiac sarcoidosis, the mean ^18^F-FDG SUVmax was significantly higher than the mean ^18^F-FLT SUVmax (*P* < 0.001).

**Conclusions:**

The results of this preliminary study suggest that ^18^F-FLT PET/CT can detect cardiac and extra-cardiac thoracic involvement in patients with newly diagnosed sarcoidosis as well as ^18^F-FDG PET/CT, although uptake of ^18^F-FLT in lesions was significantly lower than that of ^18^F-FDG. However, ^18^F-FLT PET/CT may be easier to perform since it requires neither prolonged fasting nor a special diet prior to imaging.

## Background

Sarcoidosis is a multisystem granulomatous disease of unknown etiology characterized by the presence of noncaseating granulomas in the involved organs [1]. Although it may involve any organ or system, it most commonly affects the lungs and thoracic lymph nodes, with the cardiovascular system being the third most frequently affected site [1, 2]. Although sarcoidosis has a generally favorable prognosis, 1–5% of patients die of it because of cardiorespiratory complications [1]. Therefore, early and accurate diagnosis and treatment are very important in the management of the disease.

^18^F-FDG PET is a well-established functional imaging technique for diagnostic oncologic imaging that provides data on glucose metabolism in lesions [3]. However, the technique allows the visualization not only of malignant cells but also of inflammatory cells [4, 5]. ^18^F-FDG PET has been proposed to play a role in the diagnosis and therapeutic monitoring of sarcoidosis including cardiac involvement [6–25]. The Heart Rhythm Society (HRS) and the Japanese Society of Nuclear Cardiology (JSNC) recommend using ^18^F-FDG PET for diagnosis of cardiac sarcoidosis [26, 27]. However, assessing inflammatory lesions in cardiac sarcoidosis using this modality can be challenging because ^18^F-FDG accumulates in normal myocardium, namely physiological uptake [11, 12]. Some of the main methods proposed for inhibiting increased ^18^F-FDG uptake in myocardial physiological cells include heparin injection, prolonged fasting, and dietary carbohydrate restriction before the ^18^F-FDG PET scan [11]. However, myocardial ^18^F-FDG uptake may be observed even under these conditions.

3′-Deoxy-3′-^18^F-fluorothymidine (^18^F-FLT) has been investigated as a promising PET tracer for evaluating tumor proliferative activity [28]. Zhao and colleagues in experimental rat studies reported that ^3^H-FLT uptake in granuloma was comparable to that in the tumor, whereas ^3^H-FLT uptake in turpentine oil-induced inflammation was significantly lower than that in the tumor [29]. ^3^H-FLT may accumulate in chronic granulomatous lesions with proliferative inflammation [29]. One case study of a patient with sarcoidosis showed mild ^18^F-FLT uptake and intense ^18^F-FDG uptake in lymph nodes [30]. To our knowledge, a previous report from our group was the first to describe positive findings on ^18^F-FLT PET/CT in a sarcoidosis patient with cardiac involvement [31]. In contrast to ^18^F-FDG, ^18^F-FLT uptake in the normal myocardium is low even in the absence of prolonged fasting or imposition of a special diet prior to imaging.

Limited information is available regarding the use of ^18^F-FLT in sarcoidosis. There have been no systematic studies, except the two case reports mentioned above [30, 31]. This prompted us to undertake the present study in which the uptake of ^18^F-FLT and ^18^F-FDG was compared in the evaluation of cardiac and extra-cardiac thoracic involvement in patients with newly diagnosed sarcoidosis.

## Methods

### Patients

This study was approved by our institutional ethics review board, and written informed consent was obtained from all patients.

From March 2013 to March 2017, 41 consecutive patients with known or suspected sarcoidosis underwent both ^18^F-FLT and ^18^F-FDG PET/CT studies. Patients with a history of coronary artery disease, myocarditis, valvular heart disease, cardiomyopathy of unknown etiology, or uncontrolled diabetes mellitus were excluded. Patients treated with corticosteroids and/or immunosuppressants were also excluded. Therefore, 20 patients (7 males, 13 females; mean age, 62 years; age range, 33–79 years) with clinical and/or histological diagnosis of sarcoidosis were included in this study. Cardiac sarcoidosis was defined based on the revised guidelines 2006, 2015, and 2016 for diagnosis of cardiac sarcoidosis [27, 32, 33]*.*

### Radiotracer synthesis

^18^F-FLT and ^18^F-FDG were produced using a cyclotron (HM-18; Sumitomo Heavy Industries Co.). ^18^F-FLT was synthesized using the method described by Machulla et al. [34]. The radiochemical purity of the produced ^18^F-FLT was greater than 98%. ^18^F-FDG was produced by proton bombardment of ^18^O-enriched water by a modified method of Toorongian et al. [35]. The radiochemical purity of the produced ^18^F-FDG was greater than 95%.

### PET/CT imaging

The patients fasted for at least 18 h before the ^18^F-FDG PET/CT scan. No special dietary instructions were given to patients before the ^18^F-FLT PET/CT scan. For ^18^F-FDG PET/CT, a normal blood glucose level in the peripheral blood was documented.

All acquisitions were performed using a Biograph mCT 64-slice PET/CT scanner (Siemens Medical Solutions USA Inc., Knoxville, TN, USA). Data acquisition began with CT at the following settings: no contrast agent, 120 kV, quality reference mAs, 100 mAs [using CARE Dose4D; Siemens], 0.5-s tube rotation time, 5-mm slice thickness, 5-mm increments, and pitch 0.8. PET emission scanning of the chest region with a 10-min acquisition of one-bed position was performed 60 min after intravenous injection of ^18^F-FLT or ^18^F-FDG (3.7 MBq/kg). The PET data were acquired in three-dimensional mode and were reconstructed by the baseline ordered-subsets expectation maximization bases, incorporating correction with point spread function and time-of-flight model (2 iterations, 21 subsets). A Gaussian filter with a full-width at half-maximum of 5 mm was used as a post-smoothing filter.

The 2 PET/CT scans were acquired within 4 weeks. No treatment was performed during this period.

### Data analysis

The images were visually analyzed by two experienced nuclear physicians independently using a syngo.via (Siemens Healthcare, Erlangen, Germany). Any difference of opinion was resolved by consensus. The images were evaluated for cardiac and extra-cardiac thoracic sarcoidosis. PET/CT fusion images were reviewed. For cardiac sarcoidosis, acquired images were also resliced into a series of short-axis, horizontal long-axis, and vertical long-axis images. Myocardial uptake of radiotracer was classified into four patterns: none, diffuse, focal, and focal on diffuse [13]. A focal or focal on diffuse pattern was considered as positive. A diffuse pattern was considered as inconclusive. For calculation of diagnostic accuracy, inconclusive was considered negative [36]. For extra-cardiac thoracic sarcoidosis, focal increases in radioactivity seen in locations unaccounted for by the normal biodistribution of the radiotracer were considered as positive.

For semiquantitative analysis, a volume of interest was set to identify the maximum activity within the ^18^F-FLT- or ^18^F-FDG-positive lesion in cardiac and extra-cardiac thoracic regions. Radioactivity concentrations were normalized to injected dose per patient’s body weight by calculation of standardized uptake value (SUV). The maximal SUV (SUVmax) for the lesion was calculated.

### Statistical analysis

The sensitivity, specificity, and accuracy of PET/CT with each radiotracer for diagnosing cardiac sarcoidosis were calculated. The statistical significance was determined with the Fisher exact test. Differences in semiquantitative parameters were analyzed by a Wilcoxon signed rank test. Semiquantitative data were expressed as mean ± SD. Differences were considered statistically significant at the level of *p* < 0.05.

## Results

### Patient characteristics

The clinical characteristics of the patients included in the study are shown in Table 1. Based on clinical and pathological criteria, 2 patients were diagnosed as having only cardiac sarcoidosis, 7 as having extra-cardiac thoracic sarcoidosis (3 mediastinal/hilar lymph nodes and 4 mediastinal/hilar lymph nodes and lungs), and 11 as having both cardiac and extra-cardiac thoracic sarcoidosis (9 mediastinal/hilar lymph nodes and 2 mediastinal/hilar lymph nodes and lungs).

**Table 1:** Clinical characteristics of 20 patients with newly diagnosed sarcoidosis

Patient no.	Age (years)	Sex	Selection criteria	Biopsy site	Organs involved	Myocardial uptake	
						^18^F-FDG	^18^F-FLT
1	57	F	Biopsy	LN	LN, heart	Focal	Focal
2	46	F	Biopsy	LN	LN, heart	Focal	Focal
3	71	F	Clinical diagnosis		LN, heart	Focal	Focal
4	58	F	Clinical diagnosis		LN, heart	Focal	Focal
5	75	F	Clinical diagnosis		LN, heart	Focal	Focal
6	68	M	Biopsy	LN	LN, heart	Focal	Focal
7	64	M	Biopsy	Lung, LN	LN, heart	Focal	Focal
8	76	F	Clinical diagnosis		LN, heart	Focal	None
9	70	F	Clinical diagnosis		LN, heart	Diffuse	Focal
10	52	F	Clinical diagnosis		Lung, LN, heart	Focal	Focal
11	53	F	Biopsy	Skin	Lung, LN, heart	Diffuse	Focal
12	40	M	Clinical diagnosis		Heart	Focal	Focal
13	67	M	Clinical diagnosis		Heart	Focal	Focal
14	72	M	Clinical diagnosis		LN	None	None
15	70	M	Clinical diagnosis		LN	None	None
16	57	F	Clinical diagnosis		LN	None	None
17	56	F	Biopsy	Lung	Lung, LN	None	None
18	72	F	Biopsy	Skin	Lung, LN	None	None
19	33	M	Biopsy	LN	Lung, LN	Diffuse	None
20	79	F	Biopsy	Skin	Lung, LN	Diffuse	None

*LN* lymph node

### Detection of cardiac sarcoidosis

On the visual assessment of cardiac sarcoidosis, 4/20 ^18^F-FDG scans were rated as inconclusive because the ^18^F-FDG pattern was diffuse. In contrast, no FLT scans were rated as inconclusive. Sensitivity of ^18^F-FDG PET/CT for diagnosing cardiac sarcoidosis was 85% (11/13), specificity 100% (7/7), and accuracy 90%. Sensitivity of ^18^F-FLT PET/CT for diagnosing cardiac sarcoidosis was 92% (12/13), specificity 100% (7/7), and accuracy 95%. With regard to these parameters, no significant differences were found between the ^18^F-FDG and ^18^F-FLT PET/CT studies.

In the semiquantitative assessment of cardiac sarcoidosis, the mean ± SD SUVmax for ^18^F-FDG (8.45 ± 3.50) was significantly higher than that for ^18^F-FLT (3.14 ± 0.89; *p* < 0.005).

**Fig. 1:**
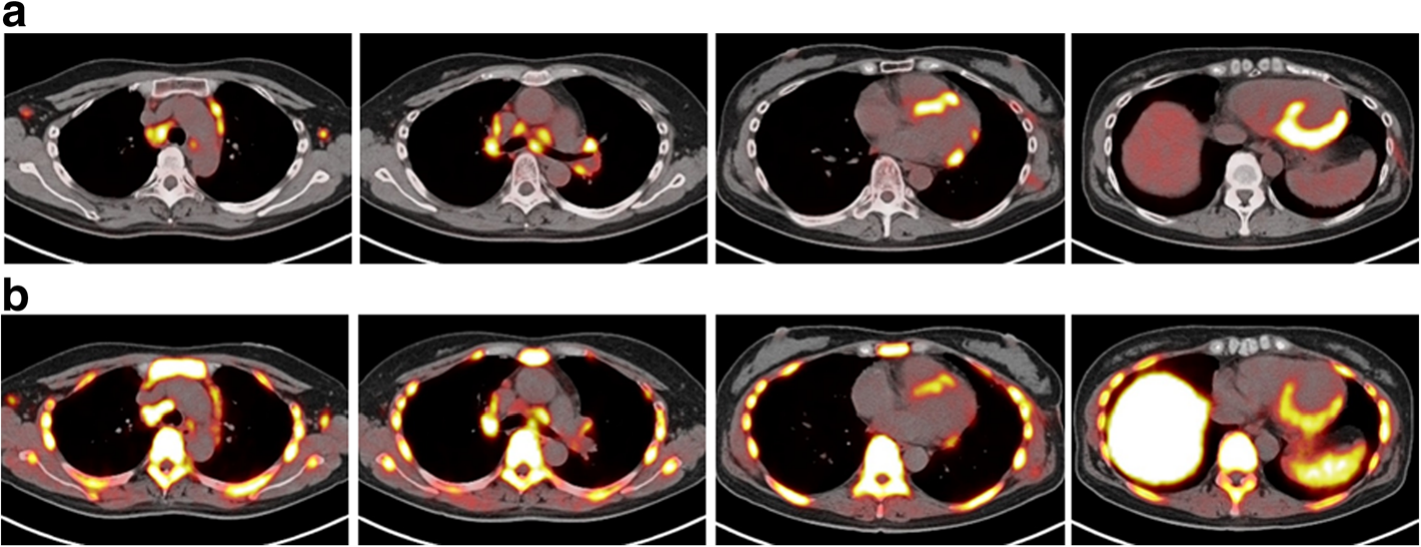
PET/CT images of a 57-year-old female with cardiac and extra-cardiac thoracic sarcoidosis (patient 1 in Table 1). Transverse ^18^F-FDG (**a**) and ^18^F-FLT (**b**) PET/CT fusion images show increased uptake in the mediastinal and hilar lymph nodes, and focal uptake at the anteroseptal, inferoseptal, and inferolateral myocardium

**Fig. 2:**
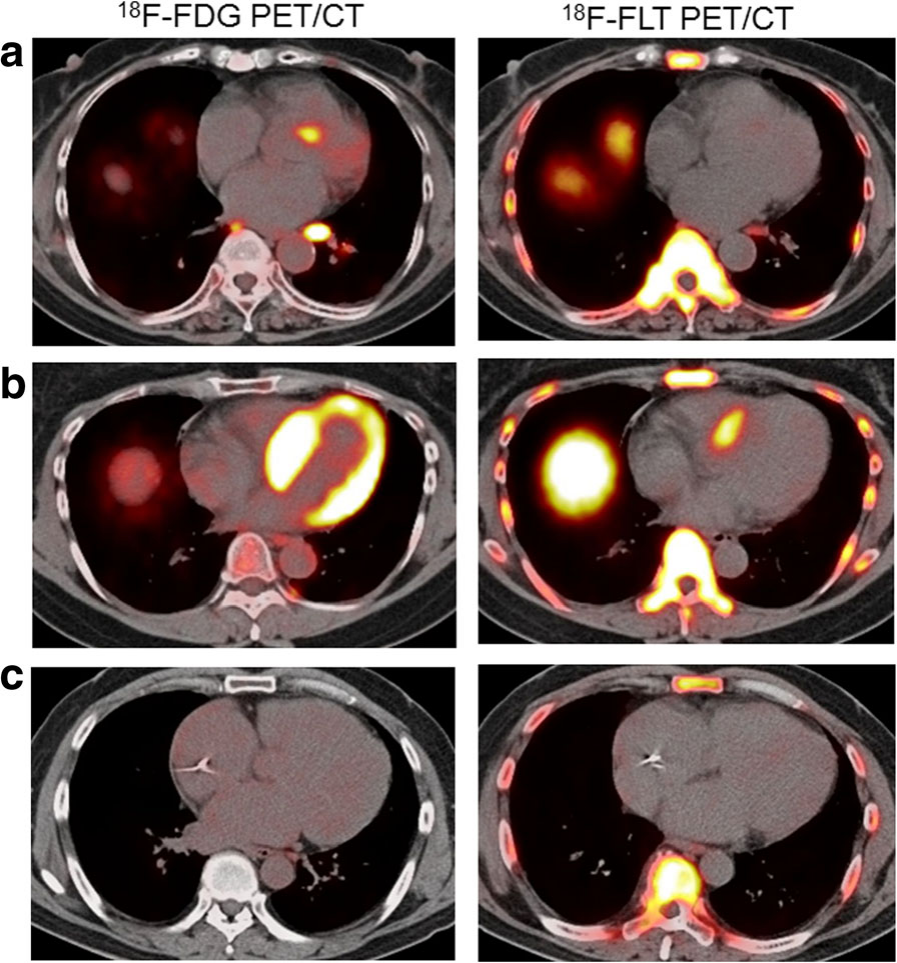
The three different patterns of cardiac uptake on transverse ^18^F-FDG and ^18^F-FLT PET/CT fusion images at cardiac level include focal ^18^F-FDG uptake in the basal anteroseptal myocardium, with no corresponding uptake of ^18^F-FLT (patient 8 in Table 1) (**a**), diffuse ^18^F-FDG uptake and focal ^18^F-FLT uptake in the upper basal anteroseptal myocardium (patient 11 in Table 1) (**b**), and no ^18^F-FDG and ^18^F-FLT uptake in the myocardium (patient 14 in Table 1) (**c**)

Figure 1 shows a typical case of newly diagnosed sarcoidosis detection by ^18^F-FDG and ^18^F-FLT PET/CT studies. Figure 2 shows 3 different patterns of cardiac uptake on ^18^F-FDG and ^18^F-FLT PET/CT studies.

### Detection of extra-cardiac sarcoidosis

On visual assessment, both ^18^F-FDG and ^18^F-FLT PET/CT studies detected all 24 extra-cardiac thoracic sarcoidosis (18 mediastinal/hilar lymph nodes and 6 lungs).

On the semiquantitative assessment of extra-cardiac thoracic sarcoidosis, the mean ± SD SUVmax for ^18^F-FDG (9.89 ± 5.07) was significantly higher than that for ^18^F-FLT (4.91 ± 2.20; *p* < 0.001).

## Discussion

In the present study of 20 patients with sarcoidosis, the detectability of cardiac and extra-cardiac thoracic involvement using ^18^F-FLT PET/CT was comparable to that using ^18^F-FDG PET/CT. In particular, for evaluation of cardiac involvement, there was no inconclusive ^18^F-FLT PET/CT scan that required any special diet prior to imaging.

^18^F-FDG PET has been used in sarcoidosis including cardiac involvement for diagnosis and therapeutic monitoring [6–25]. ^18^F-FDG PET sensitivity was reported as 97–100% for detecting extra-cardiac thoracic sarcoidosis [6–8], which is similar to the result of the present study (100%). A systematic review using ^18^F-FDG PET for detecting cardiac sarcoidosis indicated relatively high sensitivity (79–100%), which is also comparable to that of the present study (85%), but low specificity (38–100%) [9]. The present study showed a high specificity (100%). This may be attributable to inconclusive scans (diffuse pattern) that were considered negative. The reported specificity of ^18^F-FDG PET in diagnosing cardiac sarcoidosis has been varied and relatively low compared with the sensitivity [9]. One possible reason for this low specificity and high variability may be the non-specific uptake of ^18^F-FDG in the normal myocardium caused by incompletely suppressed glycolytic metabolism [9]. Some studies have shown that an 18-h fast enhanced the quality of myocardial imaging with sufficient suppression of physiological uptake [10, 11]. In the present study, although patients fasted for 18 h prior to their ^18^F-FDG PET/CT scan, 4/20 scans did not show complete suppressing of physiological ^18^F-FDG uptake. Soussan et al. reported that ^18^F-FDG PET/CT after a high-fat and low-carbohydrate diet was a sensitive tool for the diagnosis of active cardiac sarcoidosis [15]. However, the diffuse pattern or focal uptake in the papillary muscle even under such dietary stipulations was still observed in 7/30 controls and 13/58 sarcoidosis patients [15]. A recent review by Osborne et al. reported that the available literature supported use of a high-fat, no-carbohydrate diet for at least two meals with a fast of 4–12 h prior to ^18^F-FDG PET imaging and suggested that isolated fasting for less than 12 h and supplementation with food or drink just prior to imaging should be avoided [37]. Ambrosini et al. reported cardiac FDG uptake regardless of the preparation before PET/CT, suggesting that even a meal rich in fat followed by 12 h of fasting, may not be sufficient to completely suppress physiological FDG uptake [16]. Despite the proper preparation, inadequate suppression of physiological uptake in myocardium can sometimes be experienced. More easily applicable and standardized preparation protocols should be established to sufficiently suppress physiological ^18^F-FDG uptake in the normal myocardium. Another possible reason for the low specificity observed in other studies is that myocardial ischemia and heart failure may also produce focally or heterogeneously increased ^18^F-FDG uptake not related to sarcoidosis. ^18^F-FDG PET may also visualize acute myocardial inflammation to suggest active myocarditis [38]. A meta-analysis for pulmonary lesion diagnosis reported that ^18^F-FLT showed better results compared with ^18^F-FDG in ruling out inflammation-based lesions [39]. A positive ^18^F-FDG PET finding in both cardiac and extra-cardiac regions is not specific to sarcoidosis.

An ideal radiopharmaceutical for imaging cardiac sarcoidosis should have no background uptake by normal myocardial cells and not be dependent on patient diet. ^18^F-FLT PET has no physiological uptake in the myocardium and does not require patients to adhere to prolonged fasting and/or a special diet prior to imaging. However, the uptake of ^18^F-FLT in sarcoidosis has not been elucidated yet. Zhao et al. developed a rat model of granuloma characterized by epithelioid cell granuloma formation and massive lymphocyte infiltration around the granuloma, histologically similar to sarcoidosis [29]. They showed that ^3^H-FLT uptake in the granuloma was comparable to that in the tumor, as in the case of ^18^F-FDG, although the level of ^3^H-FLT uptake was lower than that of ^18^F-FDG [29]. Sarcoidosis is suggested to be a granulomatous disease with high-turnover characteristics [30]. From an in vitro study using ^3^H-thymidine, there appear to be mostly low-turnover reactions, with occasional granulomas showing high-turnover characteristics, within the lymph node of a patient with sarcoidosis [40]. Active sarcoidosis may be a granulomatous disease with high-turnover characteristics, which could account for the increased ^18^F-FLT uptake in the present study. One case study of a patient with sarcoidosis showed a mild ^18^F-FLT uptake and intense ^18^F-FDG uptake in the involved lymph nodes [30]. In the present study, uptake of ^18^F-FLT in the involved lesions was also significantly lower than that of ^18^F-FDG. To the best of our knowledge, with the exception of two sporadic case reports [30, 31], the present study is the first investigation of ^18^F-FLT PET/CT undertaken for the detection of sarcoidosis. ^18^F-FLT may be a potentially useful tracer to combine with ^18^F-FDG in the detection of sarcoidosis, especially cardiac sarcoidosis.

^18^F-sodium fluoride (NaF) similarly has little myocardial uptake and is not dependent on patient preparation in terms of diet or insulin status similar to ^18^F-FLT. Recently, Weinberg et al. investigated ^18^F-NaF PET/CT for detection of cardiac sarcoidosis in three patients [41]. However, they reported that ^18^F-NaF may not be able to effectively image active inflammation due to cardiac sarcoidosis unlike ^18^F-FDG [41]. Gormsen et al. investigated the feasibility of ^68^Ga-DOTA-NaI-octreotide (DOTANOC) PET/CT, compared with ^18^F-FDG PET/CT, for the detection of cardiac sarcoidosis [36]. They showed that the diagnostic accuracy of ^68^Ga-DOTANOC in diagnosing cardiac sarcoidosis was 100% although 11/19 ^18^F-FDG scans were rated as inconclusive despite prolonged pre-scan fasting [36]. To date, there have been few studies using newer radiopharmaceuticals other than ^18^F-FDG for evaluation of sarcoidosis.

Limitations of the present study include a small sample size and retrospective design. Only 9 patients were diagnosed histologically, while none of those with cardiac sarcoidosis were diagnosed histologically by endomyocardial biopsy. Generally, endomyocardial biopsy shows lower sensitivity due to the heterogeneous distribution of the noncaseating granulomas that are characteristic of the disease. Given the limited sensitivity of myocardial biopsy, the revised guidelines 2006 [27], 2015 [32], and 2016 [33] have been used as the diagnostic standard. An important limitation of this study is the lack of significant dietary preparation with high-fat and low-carbohydrate meals prior to the ^18^F-FDG PET studies. Further studies to compare the ^18^F-FLT PET and ^18^F-FDG PET with such a dietary preparation will be needed and are currently underway. Unfortunately, in the present study, no data on whole-body scanning were available. Teirstein et al. reported that whole-body scan with ^18^F-FDG PET was particularly useful for detecting unsuspected extra-thoracic sarcoidosis [42]. They concluded that whole-body ^18^F-FDG PET was useful mainly in the detection of occult sites for biopsy and in the assessment of the presence of residual activity in patients with fibrotic pulmonary sarcoidosis, which may help to decide whether to continue or cease steroid therapy [42]. We did not compare PET imaging and myocardial perfusion imaging. Although ^18^F-FLT PET/CT could detect cardiac and extra-cardiac thoracic sarcoidosis as well as ^18^F-FDG PET/CT, uptake of ^18^F-FLT in lesions was significantly lower than that of ^18^F-FDG. The lower level of uptake probably increases the detection limit, making it more difficult to visualize lesions. Pathology findings seen in sarcoidosis range from inflammatory cell infiltration, edema, noncaseating granuloma formation, and fibrotic changes to scarring [43]. Of these, ^18^F-FDG PET has limited ability to depict fibrous regions [24]. To date, no study has reported this issue using ^18^F-FLT PET. The combined use of ^18^F-FDG, ^18^F-FLT, other PET tracers, and other imaging modalities such as magnetic resonance imaging might be helpful in the diagnosis and staging of cardiac sarcoidosis. Additional large prospective studies are needed to determine the clinical usefulness of ^18^F-FLT PET/CT in patients with sarcoidosis.

^18^F-FDG PET can monitor the disease activity of sarcoidosis including cardiac involvement, during and after steroid therapy [7, 16–22]. However, elevations in serum glucose and insulin levels due to steroid therapy may adversely affect ^18^F-FDG uptake in target organs including the heart and reduce test specificity [5, 17]. This may preclude accurate assessment of the effects of steroids using ^18^F-FDG PET. Although ^18^F-FLT PET might be useful for avoidance of such misleading evaluations, the role of ^18^F-FLT PET in steroid therapy monitoring has not been evaluated so far. Further prospective studies involving a larger number of patients will be required to determine the clinical usefulness of ^18^F-FLT PET in the diagnosis and monitoring the effects of steroid therapy in patients with sarcoidosis.

## Conclusions

Based on the results of this preliminary study in a small patient sample, ^18^F-FLT PET/CT seems to be as effective as ^18^F-FDG PET/CT in detecting cardiac and extra-cardiac thoracic involvement in patients with newly diagnosed sarcoidosis, although uptake of ^18^F-FLT in the lesions was significantly lower than that of ^18^F-FDG. However, since ^18^F-FLT PET/CT requires neither prolonged fasting nor a special diet prior to imaging, it may be a more convenient technique for evaluation of cardiac involvement in sarcoidosis.
